# 

**Published:** 1854-11

**Authors:** 


### CONTENTS OF SECOND VOLUME.

Page.

**A**

American Journal of Pharmacy......... 80
Anatomy, notes on............................ 1
Adhesion Plaster in Fracture Patella... 39
Auscultation and Percussion............... 74
Alkursen, effects of medicine............. 8
Anæsthesia, by Cold..........................133
American Medical Profession.............137
Anæmia of Infancy...........................200
Acids in Disorder of the Bowels.........212
Alkalis in Rheumatism......................205
A Benevolent Enterprise...................311
American Medical Association............318
Autobiography of Chas. Cadwell, M. D. 315
Affections of the Cervix Uteri............374
A Circular.......................................348
Account of Cholera in Columbia.........364
Allen Prof., Notice of Appointment...343
Atkinson, M.D. on Narcotism by Opium 402
Allen Prof. on narcotics in nervous diseases.................................332—424
American Medical Association—next Meeting......................................229
Announcements ...............................479

**B**

Brain, in Cholera..............................157
Beverages in Sickness.......................182
Boston Soc. Med. Improvement.........188
Bedside Observations........................138

**C**

Cholera in Boston............................. 16
Continual fever, Iodine in.................. 14
Clinical Lecture on Whooping Cough 25
College Session................................ 72
Chloroform, questionable utility of...... 34
Case's Gutta Percha Splints.............. 18
Choleraic Diarrhœa..........................136
College Session................................152
Close of Session and Graduating Class 225
Cases Polypus Uterus.......................317
Charge of Prof. Pancoast..................315
Cases in Practice.............................321
College Announcements....................344
Chloroform in the bite of rattle snake 414
Cloroform in fevers..........................468
Cincinnati Schools ..........................466

**D**

Delerium Tremens ...........................117
Death from Old Age..........................208
Decoction of Oats as a Diuretic.........215
Dencarcotized Opium in Fevers.........211
Delegates to Amer. Med. Association...467

Page.

**E**

Egleston, case of Monstrosity............ 12
Erickson's Surgery ........................... 79
Exchanges......................................160
Epidemic Erysipelas .......................171
Ethereal Solution of Nitrate of Silver 215
Excission of Lead Bar.......................261
Error of Position..............................312
Extracts from Records of Boston Society Med. Improvements..................381
Eighth Annual Meeting of the American Medical Association.................365
Eve Prof., Valedictory......................453
Emenagogue Properties of Chamomile Flowers .......................................470

**F**

Fissures of Rectum..........................136
Formula.........................................144
Flint, Prof. Austin...........................350

**G**

Griffith's Formulary ........................ 78
Gratitude .......................................154
Gum Arabic, a substitute for............214
Gun-shot wounds of the Brain...........209
Gangrene of Lung............................471

**H**

Hughes' Prof., case in Surgery........... 20
Hughes, on Auscultation ................... 78
Hospital Reports..............................124
Hughes Prof. Operation for Necrosis...158
Hughes, Prof. of Lithotomy...............178
Homœopathy ..................................223
Hydrophobia...................................297
Humiliating ....................................306
Hydroclorate Ammonia in Neuralgia...289
Hot Douche ....................................468
Hydro-cyanate of Iron......................147
Hygiene, outraged laws of.................407
Hiccough .......................................469
History of A. M. Association.............474

**I**

Iowa Medical Society........................346
Iodine............................................136
Iodine with Chloroform.....................472
Influence of Ether............................145
Improved Stethoscope.......................216
Iowa Med. Society............................319
Illustrations of Vicarious Diseased Action.............................................258
Importance of Nourishment in Fever...285
Incontinence of Urine .....................468

Page.
Injections of water and milk in cholera 469
Iodine and Nitrate of Silver.....................469

J

Journal ........................................... 60
" ...........................................465

K

Knowles' Prof. Introductory.............. 81
Knapp, Dr. on nursing Sore Mouth 349–429

L

Luxation of Metatarsal Bones............ 37
Letter of Dr. Bowling.......................361
Leibig's Broth...................................471
Lactate of Iron in Nervous Diseases...472

M

Mental Derangement..................22—107
Medical Fogieism............................ 64
McGugin, Prof. Cases in Practice......... 97
McGugin's Prof. Report..................... 40
Medical Notes of Travel.................148
Meigs, Prof. on Child-bed Fever.........159
Medical Associations—Prize Essays...230
Medical Obituaries ...........................239
Mineral Acids in Nausea and Vomiting in Pregnancy.........................290
Medical Society in Wapello County......348

N

Nature and Treatment of Western Autumnal Diseases ...........................270
Nursing Sore Mouth, Knapp on..........349
Nitric Acid in Hooping Cough............471
New Hampshire Med. Society............475

O

Our Readers ..................................... 71
Operations for Necrosis .....................266
On the use of cold as an Anaesthetic...278
Our Journal ......................................349
Operation for Imperforate Anus .........330

P

Position in Syncope.......................... 58
Puerperal Fever ............................. 91
Pumpkin Seed .................................128
Prize Essay .....................................135
Poisoned Wounds.............................156
Professional Quackery .....................230
Positive Medical Agents ...................237
Practice of Drugging Children.........302
Poem on Rheumatism.......................309
Pulmonary Diseases, Inhalations in...296

Page.
Professional Self-Reliance—Valedictory Address...................................241
Professional Letter...........................252
Puerperal Fever, Practical Observations on.........................................337

Q

Quickness of Pulse after Fever.........282
Quack Remedy for Hydrophobia.........459

R

Ryland, Dr. on Indian Hemp.............103
Radical cure of Hydrocele..................213
Records Boston Medical Society.........292
Remedies for Disorders of Stomach...294
Rand's Med. Chemistry.....................317
Ryland, Dr. Kirtley...........................230
Rheumatism.....................................469
Ranking's Abstract...........................474

S

Scalds by Steam ..............................140
State Medical Society........................155
Surgical Clinic..................................151
Simabra Cedron ...............................156
Skin ................................................159
Surgical Instruments.........................160
Sanborn, Prof. Seventh Census .........161
" " Effects of Shaving.........202
Starling Hall ....................................312
Sanitary Commission........................313
Solidified Milk .......................288—472
Suggestions as to the Method of Using Narcotics in Nervous Diseases.........232

T

Transactions of A. M. Association ...233
Table of Urinary Deposits..................317
Treatise, Dr. Knapp's .......................463
Tirritus Aurum ................................471
Todd's Lectures on Paralysis............473
Tanner's Manual..............................478

U

Ulcerations of Os Uteri.................... 41
Urea ............................................... 53

V

Virtues of Medical Men.....................461

W

Water Dressing ...............................195
Water in Disease..............................198
Western Steamers, Sanitary Condition 217

